# The seroepidemiology of dengue in a US military population based in Puerto Rico during the early phase of the Zika pandemic

**DOI:** 10.1371/journal.pntd.0009986

**Published:** 2022-01-21

**Authors:** Simon Pollett, Caitlin H. Kuklis, David A. Barvir, Richard G. Jarman, Rachel M. Romaine, Brett M. Forshey, Gregory D. Gromowski

**Affiliations:** 1 Viral Diseases Branch, Walter Reed Army Institute of Research, Silver Spring, Maryland, United States of America; 2 Armed Forces Health Surveillance Division, Silver Spring, Maryland, United States of America; Duke-NUS GMS, SINGAPORE

## Abstract

Understanding the burden and risk factors of dengue virus (DENV) infection in Puerto Rico is important for the prevention of dengue in local, traveler and military populations. Using sera from the Department of Defense Serum Repository, we estimated the prevalence and predictors of DENV seropositivity in those who had served in Puerto Rico, stratified by birth or prior residence (“birth/residence”) in dengue-endemic versus non-endemic regions. We selected sera collected in early 2015 from 500 U.S. military members, a time-point also permitting detection of early cryptic Zika virus (ZIKV) circulation. 87.2% were born or resided in a DENV-endemic area before their military service in Puerto Rico. A high-throughput, flow-cytometry-based neutralization assay was employed to screen sera for ZIKV and DENV neutralizing antibodies, and confirmatory testing was done by plaque-reduction neutralization test (PRNT). We identified one Puerto Rico resident who seroconverted to ZIKV by June 2015, suggesting cryptic ZIKV circulation in Puerto Rico at least 4 months before the first reported cases. A further six PRNT-positive presumptive ZIKV infections which were resolved as DENV infections only by the use of paired sera. We noted 66.8% of the total study sample was DENV seropositive by early 2015. Logistic regression analysis indicated that birth/residence in a dengue non-endemic region (before military service in Puerto Rico) was associated with a lower odds of DENV exposure by January—June 2015 (aOR = 0.28, p = 0.001). Among those with birth/residence in a non-endemic country, we noted moderate evidence to support increase in odds of DENV exposure for each year of military service in Puerto Rico (aOR = 1.58, p = 0.06), but no association with age. In those with birth/residence in dengue-endemic regions (before military service in Puerto Rico), we noted that age (aOR = 1.04, p = 0.02), rather than duration of Puerto Rico service, was associated with dengue seropositivity, suggesting earlier lifetime DENV exposure. Our findings provide insights into the burden and predictors of DENV infection in local, traveler and military populations in Puerto Rico. Our study also highlights substantial PRNT ZIKV false-positivity when paired sera are not available, even during periods of very low ZIKV prevalence.

## Author summary

Understanding the risk and risk factors of dengue and Zika virus (DENV, ZIKV) infection improves their prevention. We leveraged a U.S. Department of Defense sera repository to identify sero-exposures to DENV and ZIKV in 500 adults performing military service in Puerto Rico through early 2015, the majority of who were born or previously resided in Puerto Rico. The mean duration of military service before sera were collected was 5.2 years. Serological results indicated ZIKV may have been circulating in Puerto Rico for at least 4 months before the first cases were detected in this territory. We noted ZIKV false-positivity to reference serological methods when paired sera are not available. 66.8% of the total study sample was DENV seropositive by 2015; at least 21.8% apparently had a prior single dengue infection. Among those with birth/residence in a non-dengue-endemic country, we noted an increase in the odds of DENV exposure for each year of military service in Puerto Rico. In those with birth/residence in dengue-endemic regions, we noted that age, rather than duration of Puerto Rico service, was associated with dengue seropositivity, suggesting earlier lifetime DENV exposure. Our findings provide insights into the risk and risk factors of these two important arboviruses.

## Introduction

The dengue viruses (DENVs) 1–4 are single-stranded, positive-sense RNA viruses in the family *Flaviviridae* and the genus *Flavivirus* and are primarily vectored by the anthropophilic *Aedes aegypti* mosquito [1]. Primary DENV infection provides only temporary protection against the other three DENVs and paradoxical increased risk of severe heterotypic DENV infection thereafter [2,3]. The majority of DENV infections are asymptomatic, and clinically apparent cases range from a self-limiting febrile illness to relatively uncommon manifestations such as capillary leak, shock, hemorrhage and death [4]. The mainstay of dengue therapies remains supportive treatment only, with one licensed vaccine restricted to those previously exposed to DENV [5]. DENV has close genomic and structural homology to Zika virus (ZIKV), another flavivirus that is transmitted by the *Ae*.*aegypti* and *Ae*.*albopictus* species. In 2015 and 2016, ZIKV spread rapidly and widely throughout the Americas [6,7]. Clinical manifestations of ZIKV infection range from asymptomatic or a mild febrile exanthem to fetal microcephaly, Guillain-Barre syndrome and other neurological sequelae [8,9].

Like many other dengue hyper-endemic regions in the Americas, Puerto Rico has experienced a long history of recurrent DENV epidemics which have affected local residents, travelers and military populations [10]. Puerto Rico also experienced a large ZIKV outbreak during the 2015–2017 neotropical epidemic which affected the population of Puerto Rico, travelers and US military members [11–13]. Zika cases were reported in Puerto Rico in November 2015, although it is unclear if there were earlier infections.

Understanding the prevalence and risk factors of DENV in Puerto Rico remains a key part of preparedness for future dengue outbreaks in this and other tropical regions. However, epidemiological data for DENV in Puerto Rico and other tropical settings depends primarily on the passive surveillance of medically attended febrile cases that are prone to substantial underreporting, case definition heterogeneity, and sampling bias [11,12,14–17]. While periodic outbreak household cluster investigations, a robust passive dengue surveillance system, and enhanced sentinel surveillance systems are part of the Puerto Rico public health structure, these typically miss the majority of asymptomatic or minimally symptomatic DENV cases [11,18–20]. During outbreaks of ZIKV, such surveillance systems may also conflate ZIKV and DENV cases due to overlapping clinical symptoms and signs, compounded by substantive cross-reactivity of many ZIKV and DENV serological assays used in clinical care and public health, even though ZIKV falls outside the DENV sero-complex [21–23].

Longitudinal serological cohorts have offered major insights into the incidence and risk factors of asymptomatic and symptomatic DENV infections in Puerto Rico other tropical regions in the Americas and Asia, but these are typically limited to pediatric cases and are resource intensive [15,24–27]. Another limitation of these sero-studies is that they cannot directly compare the risks and frequency of travel-related DENV infections (including those in deployed U.S. military) with those in the local resident population. Valuable studies on the epidemiology of DENV-infected traveler populations have been performed, but are either restricted to often small serological studies with short term travelers, or–more commonly–febrile surveillance studies of returning travelers, which miss many incident DENV cases during travel [28–30].

Drawing from a unique US military sera repository of 64 million sera samples, we undertook a DENV sero-epidemiology study in those who had performed U.S. military service in Puerto Rico through early 2015. Specifically, we compared the prevalence and predictors of DENV infection in those U.S. military members born or residing in a dengue-endemic country versus those born or residing in non-dengue-endemic countries before their Puerto Rico service. By selecting an early 2015 sampling time point, we were also able to test for local cryptic ZIKV circulation before the reported first cases in November 2015. We further used antecedent paired sera from ZIKV seropositive cases to confirm exposures and examined the levels of ZIKV false positivity with a reference plaque-reduction neutralizing test during a known period of very low ZIKV prevalence.

## Methods

### Ethics

The study was approved by the Defense Health Agency Human Research Protection Office (CDO-16-2017). Consent was not obtained as this was considered non-human subjects research using deidentified sera and metadata collected for operational purposes (HIV screening) and already available in the DODSR for sero-epidemiological studies.

### Study sample and study population

Sera specimens were selected from the US Department of Defense Serum Repository (DODSR) that consists of sera collected from active duty and reserve components of the U.S. military from routine biennial HIV blood screening and as part of required pre- and post-deployment blood draws [31]. Serum specimens were identified from 500 randomly selected active duty, guard, or reserve military members who were assigned to Puerto Rico for a period of time overlapping with a one-year window between April 2014 and April 2015 and had a DODSR serum sample drawn between 01 January 2015 and 01 June 2015. We excluded those subjects who were in Puerto Rico for less than 30 days before their sera was taken. This sampling time preceded the first known cases of ZIKV in Puerto Rico in November 2015 [11]. To confidently discriminate ZIKV from DENV infections, we also selected an antecedent sera specimen for each subject, preceding January 01 2015, to be used to exclude ZIKV false seropositivity.

All sera specimens were accompanied by data on the subject’s sex, age, country/territory of birth, country/territory of residence (at time of commencing military service), years of Puerto Rico deployment/activation (before the 2015 sera collection), branch of military service, rank (officer versus enlisted), and role/status (active duty, reserve, guard) which were collected as part of the routine DODSR banking process. We classified whether the subject was born or resided in a dengue endemic (versus a dengue non-endemic) region prior to Puerto Rico military service by searching for that country or territory in CDC travel advisory database of regions with frequent or continuous dengue risk [32].

### Laboratory methods

#### Overview

We employed a high-throughput, flow cytometry-based neutralization (FlowNT) assay to screen serum samples for ZIKV and DENV neutralizing antibody (NAb) [33,34]. Samples that demonstrated >80% neutralization of ZIKV infectivity at a 1:40 FlowNT screening dilution were subsequently screened at a 1:10 dilution using a standard plaque reduction neutralization (PRNT) assay. To exclude false ZIKV seropositivity, all ZIKV positive sera by FlowNT and PRNT had PRNT50 titers also performed on antecedent sera to exclude DENV cross-reactivity.

#### Viruses

The virus strains used for the both the FlowNT and the PRNT assays were DENV-1 West Pac 74, DENV-2 S16803, DENV-3 CH53489, DENV-4 TVP-360, and ZIKV Paraiba_01.

#### Flow cytometry-based neutralization (FlowNT) assay

Sera were heat inactivated for 30 min at 56 degrees C prior to testing. A 1:40 dilution of serum was mixed with an equal volume of virus and incubated for 1 hr at 37 degrees C, followed by infection of U937-DC-SIGN cells. At 24 hrs post infection, cells were fixed, permeabilized and immunostained. The percent infected cells was determined by flow cytometry. Serum that neutralized ≥ 80% of virus infectivity were considered seropositive for that virus.

#### Plaque reduction neutralization (PRNT) assay

Serum dilutions were mixed with an equal volume of virus and incubated for 1 hr at 37 degrees, followed by infection of Vero cells. Plaques were detected by staining with neutral red. Screening was performed at a 1:10 dilution of serum and ≥ 50% reduction of plaque forming units (PFU) was considered seropositive. The PRNT titers were determined by testing serial dilutions of sera starting at 1:20 and the 50% neutralization antibody (NAb) titers were determined by non-linear regression. For paired sera specimens tested, a ≥ 4-fold rise in PRNT50 titer among paired samples was interpreted as a seroconversion to the respective virus.

#### Statistical analysis

Subject demographic and professional characteristics were summarized as means or proportions with 95% confidence intervals. ZIKV and DENV seropositivity estimates were presented with 95% CI, and further stratified by whether the subject was born or resided (“birth/residence”) in dengue endemic (versus a dengue non-endemic) region prior to Puerto Rico military service [32]. Independent predictors of DENV seropositivity were determined with a logistic regression model fit with sex, age, birth/residence in a dengue-endemic country, years of Puerto Rico military service before sampling, military service branch, rank (officer versus enlisted) and role (active duty, reservist, guard). Model fit was determined by Akaike information criteria (AIC) and Hosmer-Lemeshow goodness-of-fit tests. All statistical analyses were performed by Stata v15.1 (Statacorp, College Station, TX, USA).

## Results

Of the n = 500 DODSR sera selected, n = 6 subjects were excluded (were in Puerto Rico for less than 30 days before their sera was taken, or sera actually preceded the time of Puerto Rico assignment commencement). The mean age of the n = 494 final study sample was 28.0 years and 84.0% were male. The mean time of military service in Puerto Rico prior to the January-June 2015 sera collection was 5.2 years (Table 1). The study sample comprised of two distinct study populations. The first was those born in or residing in a DENV endemic country or territory before Puerto Rico military service (87.2%), primarily birth/residence in Puerto Rico, but also those with a birth country which included Argentina (n = 1), Colombia (n = 1), Cuba (n = 1), Dominican Republic (n = 8), Ecuador (n = 1), Panama (n = 1), Peru (n = 1), Philippines (n = 1), Venezuela (n = 1), Vietnam (n = 2), and Virgin Islands (n = 1). The second population was those born and residing in a non-DENV endemic country or territory before their Puerto Rico military service (principally continental USA). The high proportion of U.S. military members born in dengue-endemic countries (especially Puerto Rico) in this sample was a reflection of most military service within Puerto Rico being performed by local residents who activate as Guard or Reservists (Table 1).

**Table 1:** Demographic and professional characteristics of n = 494 US military assigned to Puerto Rico and sampled January—June 2015.

	%
Male gender	84.0
Military service	
Army	83.8
Coast Guard	10.5
Navy	3.2
Airforce	1.6
Marine	0.8
Military rank	
Enlisted	90.7
Officer	9.3
Active duty status	
Active duty	12.3
Guard or reserves	87.7
Born and/or resided in a dengue endemic country*	87.2
Race & ethnicity	
Asian, Pacific Islander	0.8
Black, Non-Hispanic	1.2
White, Hispanic	88.7
White, Non-Hispanic	8.1
Other or Unknown	1.2
	Mean (range)
Age (years)	27.9 (17–55)
Years deployed/activated in Puerto Rico	5.2 (0.2–15.6)

*Before onset of Puerto Rico assignment

**Fig 1:**
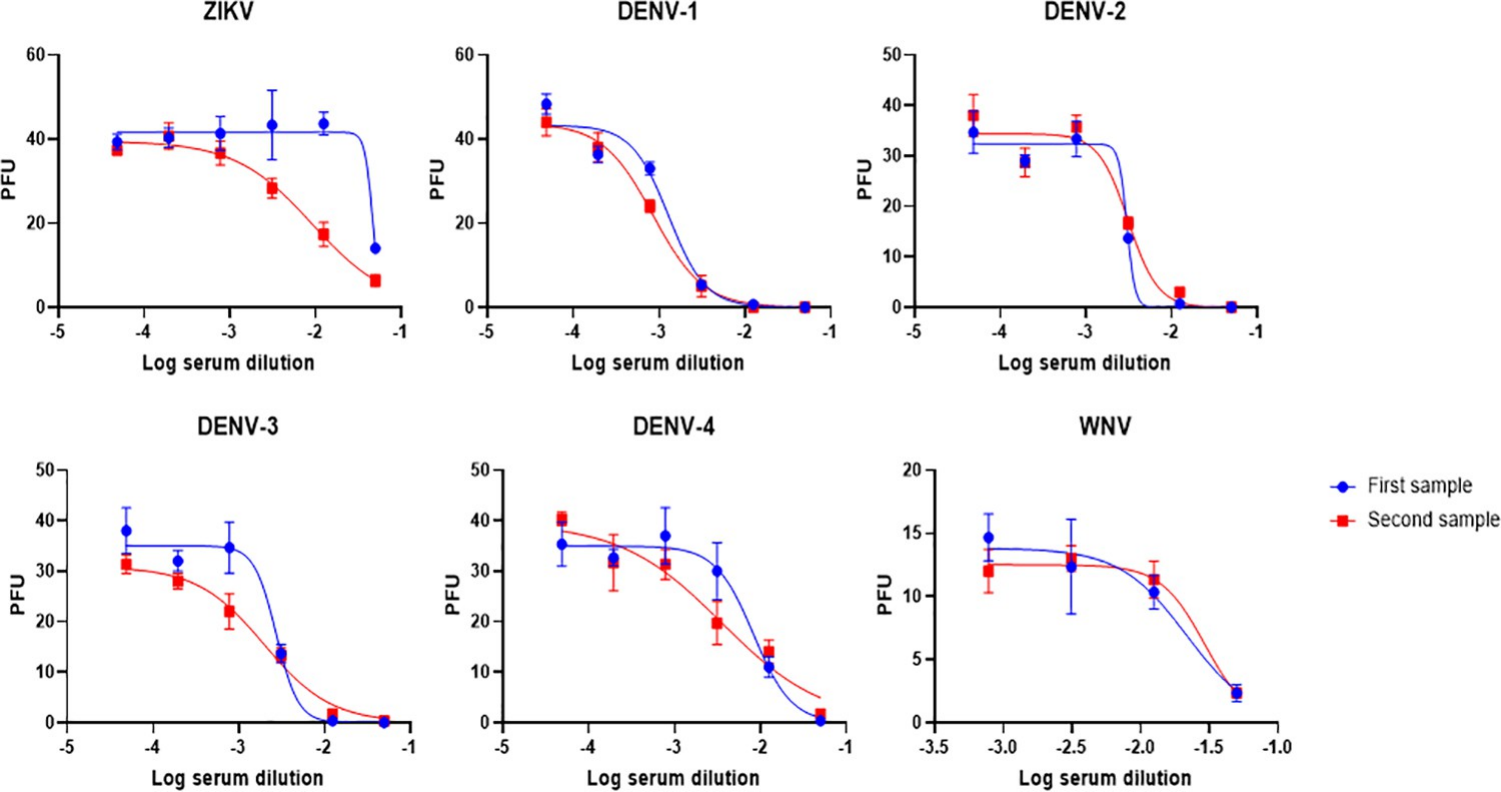
Subject PR140 neutralization curves for ZIKV, DENV 1–4, and WNV.

We noted 33.0% (163/494) had detectable ZIKV NAb by FlowNT screening. However, only 1.6% (7/494) individuals had detectable ZIKV NAb by both FlowNT and PRNT screening assays (Table 2). The 50% neutralization titers were determined for paired sera in these seven subjects to delineate true ZIKV seroconversions from DENV cross-reactivity (Table 3). We noted that 6 of 7 of these single timepoint sera that demonstrated >50% ZIKV neutralizing activity by PRNT at a 1:10 screening dilution were actually DENV exposed individuals that had some cross-reactive ZIKV NAb, but did not have evidence of seroconversion when paired sera were analyzed (Table 3). Using this paired specimen approach we confirmed that only one individual seroconverted to ZIKV during this time period (in the absence of DENV or WNV seroconversion) by June 2015 (Table 2 and Fig 1), an adult male that was born and resided in Puerto Rico before his military service in that territory.

**Table 2:** Seropositivity to dengue virus and Zika virus in 494 US military assigned to Puerto Rico and sampled Jan 01 2015—June 01 2015.

	% Seropositive, 95% CI		
	Born/resided in dengue endemic country prior to deployment (N = 429)	Born/resided in dengue non-endemic country prior to deployment (N = 63)	All (N = 494)^e^
DENV seropositive^a^	72.7% (68.3–76.9)	28.6% (17.9–41.3)	66.8% (62.5–70.9)
DENV primary infection^b^	23.7% (19.8–28.1)	9.5% (3.6–19.6)	21.8% (18.3–25.8)
ZIKV seropositive, suspected^c^	1.6% (0.6–3.3)	0% (0–5.7^f^)	1.4% (0.6–2.9)
ZIKV seropositive, confirmed^d^	0.2% (0.01–1.3%)	0% (0–5.7^f^)	0.2% (0.01–1.1)

^a^NT80 positive by FlowNT to any serotype

^b^Monvalent positivity by FlowNT, excludes multivalent responses of which some may be a primary infection with cross-serotype reactivity

^c^PRNT to ZIKV and DENV on a single sera specimen suggestive of ZIKV positivity

^d^PRNT seroconversion to ZIKV (and not DENV) based on paired antecedent sera specimen

^e^Birth/residence status of n = 2 subjects unknown

^f^One-sided 97.5% confidence interval

**Table 3:** Neutralizing antibody titers for ZIKV, DENV 1–4, and West Nile virus for cases positive for ZIKV by PRNT and FlowNT.

Subject	First sample PRNT50^a^						Second sample PRNT50^b^						NT50 fold-change					
	ZIKV	D1	D2	D3	D4	WNV	ZIKV	D1	D2	D3	D4	WNV	ZIKV	D1	D2	D3	D4	WNV
PR266	22	1684	1826	6536	369	NT	29	327	302	2082	328	NT	1.3	0.2	0.2	0.3	0.9	NA
PR445	<20	37	66	370	91	NT	~10^c^	35	52	334	80	NT	1	0.9	0.8	0.9	0.9	NA
PR420	<20	183	65	20	31	NT	20	287	170	61	81	NT	1.0	1.6	2.6	3	2.6	NA
PR093	<20	533	675	499	858	NT	~10^c^	505	599	240	454	NT	1	0.9	0.9	0.5	0.5	NA
PR140	22	772	331	365	121	46	109	1176	321	504	183	34	5.1	1.5	1	1.4	1.5	0.7
PR456	44	843	505	686	84	NT	30	329	410	678	61	NT	0.7	0.4	0.8	1	0.7	NA
PR078	<20	131	129	450	127	NT	~10^c^	136	78	525	148	NT	1	1	0.6	1.2	1.2	NA

^a^Sera specimen taken before January 01—June 01 2015

^b^Sera specimen taken during January 01—June 01 2015

^c^Sample was ZIKV positive by PRNT screening at a 1:10 dilution using a 50% neutralization cutoff.

**Fig 2:**
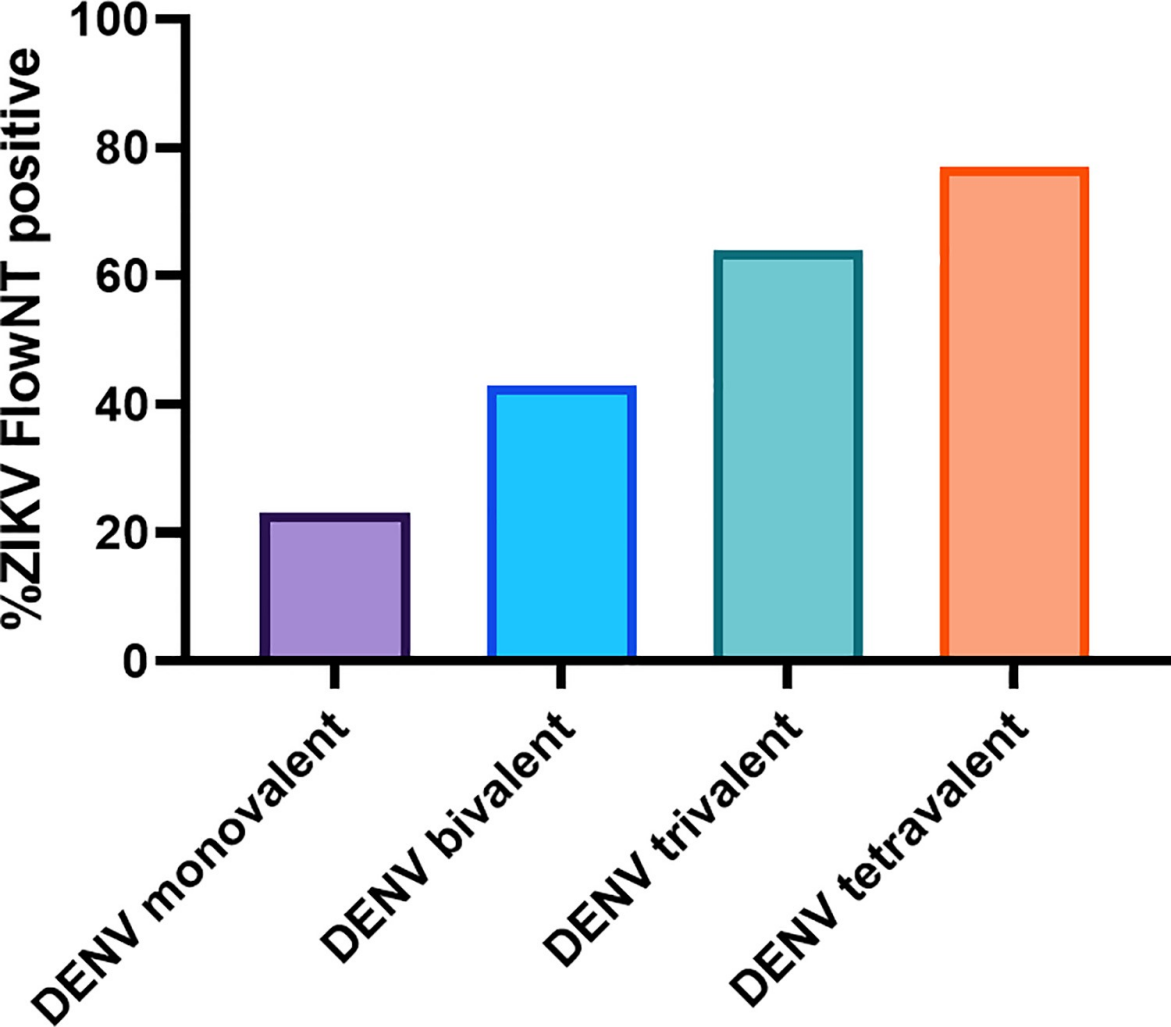
Frequency of FlowNT ZIKV seropositivity by DENV sero-valency.

The DENV seroprevalence was high in this study sample (Table 2), with 66.8% (95% CI = 62.5–70.9%) of the study sample seropositive to DENV. This was substantially higher in those with birth/residence in a dengue endemic country or territory, compared to those with birth/residence in a non-dengue endemic country (before their Puerto Rico military service). 21.8% of the study sample had detectable NAb to only a single DENV serotype (Table 2), consistent with DENV primary infections. The remainder of DENV seropositive subjects had neutralizing antibody responses to more than one serotype. Among this latter group it was challenging to distinguish primary versus secondary (or post-secondary) infections because of possible cross-reactive antibodies elicited from a recent primary infection. Taken together, the number of primary infections noted among DENV seropositive subjects in Table 2 is likely to be an underestimate. Among DENV seropositive subjects, we noted that cross-reactive ZIKV NAb seropositivity measured by FlowNT screening correlated with DENV NAb valency (Fig 2).

Logistic regression analysis indicated that birth/residence in a non-dengue endemic region before military service was associated with a reduction in the odds of being dengue seropositive (aOR = 0.28, 95% CI = 0.13–0.61, p = 0.001) (Table 4). Each year of military service in PR was associated with a 7% increase in the odds of DENV seropositivity (OR 1.07, 95% CI = 1.01–1.13, p = 0.02) (Table 4). However, as seroconversion risk over deployment time in Puerto Rico may be strongly influenced by prior DENV exposure risk and age, interpretation of this risk estimate was challenging. Indeed, inclusion of an effect modification term of birth/residence in non-DENV endemic country and years of Puerto Rico military service was statistically significant (p-value = 0.04) and improved model fit (AIC = 560.2). We therefore repeated the logistic regression analysis on the study sample stratified by birth/residence in dengue endemic versus non-DENV endemic regions (Table 5). When restricted to those born/residing in dengue endemic regions, we noted age was associated with DENV seropositivity (aOR = 1.04, 95% CI = 1.01–1.07), but years of Puerto Rico service was no longer statistically significant (Table 5). In those not born or residing in a DENV endemic country prior to Puerto Rico service, there was moderate evidence that duration of Puerto Rico service increased the odds of DENV seropositivity (aOR = 1.58, 95% CI 0.98–2.56), but age was not associated with DENV seropositivity (aOR = 1.02 (0.93–1.11), p = 0.71). This stratified analysis also noted moderate evidence for an association between Army service and DENV seropositivity in the group born/residing in a dengue endemic country before Puerto Rico military service (aOR = 2.19, p = 0.07), but the association between sex and DENV exposure was no longer statistically significant in either population (Table 5).

**Table 4:** Predictors of DENV seropositivity of n = 494 US military assigned to Puerto Rico and sampled January 01 2015—June 01 2015.

Covariate	Crude odds ratio (95% CI)	p-value	Adjusted odds ratio^a^	p-value
Male gender	0.59 (0.34–1.03)	0.062	0.55 (0.30–1.01)	0.058
Age	1.01 (0.98–1.03)	0.497	1.03 (1.00–1.06)	0.033
Born/resided in non-dengue endemic country^b^	0.15 (0.08–0.27)	<0.001	0.28 (0.13–0.61)	0.001
Years of Puerto Rico assignment before sampling	1.13 (1.07–1.19)	<0.001	1.07 (1.01–1.13)	0.02
Army (versus other Service)	4.68 (2.83–7.76)	<0.001	1.60 (0.74–3.46)	0.232
Active duty (versus Guard or Reserves)	0.13 (0.07–0.25)	<0.001	0.47 (0.18–1.21)	0.117
Officer (versus enlisted)	0.83 (0.44–1.56)	0.57	1.26 (0.58–2.76)	0.557

^a^Model includes all seven covariates, AIC = 565.3, Hosmer-Lemeshow goodness-of-fit p = 0.48

^b^Before assignment in Puerto Rico

**Table 5:** Predictors of DENV seropositivity of n = 494 US military assigned to Puerto Rico and sampled January 01 2015—June 01 2015, stratified by birth/residence in dengue endemic vs non-dengue endemic regions.

	Born/residing in dengue endemic country before deployment		Not born/residing in dengue endemic country before deployment	
	Adjusted odds ratio^a^	p-value	Adjusted odds ratio^a^	p-value
Male gender	0.57 (0.30–1.10)	0.10	0.42 (0.07–2.41)	0.33
Age	1.04 (1.01–1.07)	0.02	1.02 (0.93–1.11)	0.71
Years of Puerto Rico deployment	1.05 (0.99–1.10)	0.12	1.58 (0.98–2.56)	0.06
Army (versus other service)	2.19 (0.92–5.19)	0.07	0.47 (0.03–6.58)	0.58
Active duty (versus Guard/Reserves)	0.64 (0.19–2.15)	0.47	0.20 (0.01–3.02)	0.25
Officer (versus enlisted)	1.66 (0.62–4.41)	0.31	0.78 (0.13–4.76)	0.79

^a^Model includes all six covariates

## Conclusions

Here, we leverage a unique repository of sera from US military members (DODSR) to estimate the prevalence and predictors of DENV infection during Puerto Rico military service, with a direct comparison in those with and without birthplace/residence in DENV endemic regions prior to military service, thereby examining how prior DENV exposure modifies DENV infection risk and risk factors during adulthood. To our knowledge this is the first such study, and our findings offer insights into the epidemiology of dengue in Puerto Rico, in other dengue-endemic populations, and in traveler and military populations. This study also provides important ZIKV sero-diagnostic insights, provides estimates on the earliest circulation of ZIKV in Puerto Rico, and emphasizes the unique ability of the DODSR to answer important questions about the epidemiology of emerging viruses.

We noted that cross-reactive ZIKV NAb seropositivity by FlowNT correlated with DENV NAb valency (Fig 2), which may reflect more recent DENV exposure and a heterotypic NAb repertoire. This is consistent with prior studies which have noted that ZIKV PRNT and other assays have the greatest cross-reactivity to DENV relatively soon after DENV infection [22,35,36]. The PRNT assay appeared to have better specificity than FlowNT for detecting ZIKV NAb in sera from individuals that were DENV seropositive. The major biological difference is the cell types used in the PRNT (Vero cells) and FlowNT (U937-DC-SIGN cells). It is possible that a cross-reactive NAb epitope that is shared between DENV and ZIKV is particularly effective at blocking attachment of ZIKV to DC-SIGN, which is for the most part required for infection of U937 cells. Vero cells do not express DC-SIGN and therefore ZIKV uses alternative attachment factors. Nevertheless, we noted that six of the seven subjects who were deemed ZIKV seropositive by PRNT at a single time point were confirmed as DENV exposures when paired sera where used. These findings underscore how ZIKV PRNT false positivity may be substantive when only a single specimen used, as noted in other studies [22,37]. This highlights the importance of paired serological specimens to best characterize the burden of Zika in dengue-endemic regions, and to estimate dengue prevalence in regions which have experienced prior Zika outbreaks, as is currently the case in much of the Americas [22,36].

We note ZIKV may have been circulating in Puerto Rico for at least 4 months before the first cases were recognized and reported in November 2015 [11]. This estimate of cryptic (“silent”) circulation is consistent with evolutionary estimates from genomic sequence data sampled in Puerto Rico [38,39]. This finding highlights how ZIKV may circulate in a population for some time before detection by medically attended and sentinel surveillance systems.

We noted a high DENV seropositivity rate (66.8%) in this US military population who had performed service in Puerto Rico, with a substantial number (21.8%) having a NT80 profile possibly consistent with a single serotype infection history. Among these DENV seropositive cases, the number of primary infections were likely underestimated due to the difficulty in serologically distinguishing primary and secondary infection. This may indicate a large number of this US military subgroup may be primed for a second, more severe DENV infection via antibody dependent enhancement upon future deployments [3]. We noted a seroprevalence of 72.7% (95% CI, 68.3–76.9) in those who were born/resided in Puerto Rico before their military service, exceeding the estimated seroprevalence in younger Puerto Rico residents age 2–16 (55.9%,) by L’Azou et al [40].

We observed lower DENV seropositivity in those with birth/residence in non-DENV endemic regions before their Puerto Rico service. Nevertheless, the 28.6% (95% CI, 17.9–41.3) seropositivity rate in this group exceeded that of several other studies of the US military, including an estimated seroprevalence (9.6%) of US Special Forces with a history of tropical deployments, and a seroprevalence of 7.6% in US Service members who had undertaken a single deployment to a dengue endemic region. This seropositivity rate also exceeded the PRNT-confirmed seroprevalence (12%) of a US study population that had traveled or lived in DENV endemic countries [28,41–43]. This seropositivity rate was also considerably higher than other traveler sero-studies which included typically short-term travel (these attack rates ranged from 4.4–6.8%)[28,29,44,45]. This high seropositivity rate may reflect the long period of time spent in Puerto Rico by these U.S. military members (2.78 mean years), which is longer than typical U.S. military deployments and civilian travel. This estimate may also be skewed by several limitations of this study. First, a proportion of these subjects may have received another flavivirus vaccine such as the YFV or JEV vaccine, although the stringent FlowNT NT80 cut-off should have minimized cross-reactivity and there is little to no cross neutralization of DENV 1–4 with sera from individuals vaccinated or exposed to JEV and YFV [46–49]. Second, some of these subjects may have deployed to other dengue endemic regions before their time in Puerto Rico. Third, we did not test paired specimens before and after subject time in Puerto Rico to confirm that incident infection specifically occurred during time in this country.

We showed that birth in a DENV endemic region is an important effect modifier on the risk of DENV infection over time during military service in Puerto Rico (Table 5). In those born in DENV endemic region, age–but not duration of Puerto Rico military service—was associated with DENV seropositivity, consistent with DENV exposure occurring earlier in their life. In those US military members who were not born/residing in a DENV endemic country before their Puerto Rico service, we found that age was not associated with seropositivity, but that the odds of DENV seropositivity increased by 58% for each year of Puerto Rico military service (Table 5), although there was borderline statistical significance for this finding (p = 0.06). This association between deployment time and seropositivity in this military subgroup is consistent with traveler serostudies which have shown DENV seropositivity is associated with years lived in a DENV endemic area or the number of trips to a DENV endemic area [43,50,51]. A study of DENV serocoversions in US military first-time deployments by Hesse et al. did show a higher deployment duration in DENV seroconverters vs non-converters but this was not statistically significant (p = 0.15), although a multivariate analysis was not used, and this study sampled typically shorter durations of deployments [42].

We noted that in those who were born or resided in a DENV endemic region (mostly Puerto Rico) before their Puerto Rico military service, there was moderate evidence that Army service was an independent of DENV seropositivity after multivariate adjustment (Table 5, aOR = 2.19, p = 0.07). The association between Army service and DENV seroposivity (compared to other US military services) was also seen by Hesse et al. in their analysis of DENV seroconversions in U.S. military members deploying to DENV endemic countries in the Americas, Asia and Africa [42]. The reason for this is unclear. It may reflect an increased exposure to mosquito vectors based on occupation specialty and should be studied further.

One limitation of this study is that a single time-point was analyzed for the majority of specimens, meaning we were unable to explicitly confirm DENV seroconversions across their Puerto Rico military service. Still, by modeling deployment time and age, we were able to infer infection histories in this population. In addition, by using paired specimens for those PRNT positive by ZIKV and DENV, we were able to discriminate between ZIKV and DENV exposures. An important caveat for the field is that some neutralization assays may conflate exposures to DENV and ZIKV, even during periods of low ZIKV prevalence.

Taken together, our findings emphasize the value of serosurveillance in evaluating the risk and risk-factors of DENV and ZIKV exposure to endemic, traveler and military populations, and prompts further studies that leverage archived sera available from the DODSR to reconstruct the epidemiology of these and other arboviruses in other tropical locations.

## Supporting information

S1 Table DENV and ZIKV serological data and data dictionary. (XLS)
